# Best Practices in Adolescent and Young Adult Patients with Acute Lymphoblastic Leukemia: A Focus on Asparaginase

**DOI:** 10.1089/jayao.2015.0014

**Published:** 2015-09-01

**Authors:** Nicolas Boissel, Leonard S. Sender

**Affiliations:** ^1^ Department of Adult Hematology, Saint-Louis Hospital, University of Paris, Paris, France.; ^2^ Department of Epidemiology, University of California, Irvine, Irvine, California.; ^3^ Chao Family Comprehensive Cancer Center, University of California, Irvine, Irvine, California.; ^4^ Hyundai Cancer Institute, CHOC Children's Hospital, Orange, California.

**Keywords:** acute lymphoblastic leukemia, asparaginase, young adult, adolescent

## Abstract

The inclusion of asparaginase in chemotherapy regimens to treat acute lymphoblastic leukemia (ALL) has had a positive impact on survival in pediatric patients. Historically, asparaginase has been excluded from most treatment protocols for adolescent and young adult (AYA) patients because of perceived toxicity in this population, and this is believed to have contributed to poorer outcomes in these patients. However, retrospective analyses over the past 12 years have shown that 2-, 5-, and 7-year overall survival of AYA patients is significantly improved with pediatric versus adult protocols. The addition of asparaginase to adult protocols yielded high rates of first remission and improved survival. However, long-term survival remains lower compared with what has been seen in pediatrics. The notion that asparaginase is poorly tolerated by AYA patients has been challenged in multiple studies. In some, but not all, studies, the incidences of hepatic and pancreatic toxicities were higher in AYA patients, whereas the rates of hypersensitivity reactions did not appear to differ with age. There is an increased risk of venous thromboembolic events, and management with anti-coagulation therapy is recommended. Overall, the risk of therapy-related mortality is low. Together, this suggests that high-intensity pediatric protocols offer an effective and tolerable approach to treating ALL in the AYA population.

Acute lymphoblastic leukemia (ALL) is a heterogenic disease that disproportionately affects children between the ages of 2 and 10 years.^1–3^ Roughly 6000 new cases of ALL are diagnosed each year in the United States alone.^2,4^ Substantial advancements have been made in crafting effective multiagent pediatric chemotherapeutic protocols, raising overall survival (OS) rates for pediatric patients to nearly 90%.^5,6^ Unfortunately, there has been less success in achieving these same improvements in adolescent and young adult (AYA) patients diagnosed with ALL.^7^ In contrast to the high survival rates in pediatric patients, 5-year survival rates for adolescents (aged 15–19 years) and young adults (aged 20–29 years) diagnosed with ALL are approximately 61.1% and 44.8%, respectively (1973–2004 limited use database of the Surveillance, Epidemiology, and End Results Program of the U.S. National Cancer Institute issued in April 2007).^7^

Poor outcomes in AYA patients diagnosed with ALL likely result from a number of factors. AYA patients display a greater incidence of adverse prognostic indicators, including an increased prevalence of adverse genetic abnormalities, compared with younger patients.^8,9^ Several socioeconomic factors, such as reduced enrollment in clinical trials, possibly lower compliance rates, and increased delays in treatment initiation, may also contribute to the poor outcomes seen in the AYA population.^10^ Arguably, one of the most important factors in the disproportionate survival between pediatric and AYA patients are the chemotherapy protocols historically used to treat these two groups of patients. Most pediatric ALL protocols follow the Berlin-Frankfurt-Munster (BFM) model with a heavy reliance on nonmyelosupressive agents (vincristine, steroids, and asparaginase).^11^ Adult protocols were for a long time characterized by a greater use of myelosupressive agents (including cyclophosphamide and anthracyclines) with little or no asparaginase, as the enzyme was perceived to be prohibitively toxic in adults.^12^ In fact, one widely used adult ALL protocol, hyper-CVAD, includes no asparaginase.^13^ Recently, a wealth of clinical evidence has emerged challenging the differential treatment of pediatric and AYA patients with ALL.^14–21^ Retrospective studies report that high-intensity pediatric protocols are both feasible and tolerable in patients aged ≥15 years and that the use of these protocols is associated with substantially improved long-term survival compared with commonly used adult protocols.^14,22–24^ This review will summarize the relevant differences between pediatric and AYA leukemia biology and treatment, highlighting the rationale for treating adult patients according to pediatric protocols. The use of asparaginase, a near-universal component of pediatric ALL regimens, will be reviewed in detail.

## Disease Biology

There are a number of biological factors that contribute to low cure rates in AYA patients. T-cell ALL is known to be associated with poor outcomes and is seen in 20–25% of adult cases of ALL compared with 15% in children.^15,25^ Furthermore, early T-cell precursor leukemia is a particularly high-risk subtype of T-cell ALL found in approximately 15% of T-cell ALL. This subtype has been characterized by a high risk of treatment failure and poor prognosis in patients.^26,27^ However, recent results in larger patient populations have shown non-inferior outcomes in patients with T-cell and early T-cell precursor ALL.^28^ Several chromosomal abnormalities associated with negative outcomes are more commonly observed in adult patients relative to pediatric patients with ALL.^29^ The incidence of Philadelphia chromosome positive (Ph1) ALL, strongly associated with poor outcomes, is one of the most common cytogenetic abnormalities in adult ALL.^30^ Ph1 ALL is found in 15–20% of patients aged 25–35 years compared with <3% of patients aged <18 years.^23,30,31^

Further compounding the problem, genetic alterations associated with positive prognosis occur less often in AYA patients. High hyperdiploidy, associated with favorable prognosis, occurs in 25–30% of pediatric patients with ALL, but only in 20% of adolescents and 10% of young adult patients.^23^ Similarly, the TEL-AML1 fusion gene occurs in approximately 25% of pediatric cases compared with just 3% of adults with ALL.^32^ Other adverse prognostic features, such as intrachromosomal amplification of chromosome 21, *MLL* translocations, and *IGH@* translocations, have been shown to occur more frequently in the AYA population.^26,33,34^ Ph1-like ALL is a subtype of B-cell ALL with similar patterns of gene expression to Ph1 ALL, but does not express the BCR-ABL1 fusion protein.^35,36^ Ph1-like ALL has been reported in 27% of B-cell ALL patients between 21 and 39 years of age, compared with 10% of patients between 1 and 9 years of age.^36^ This subtype has been associated with a resistance to asparaginase and daunorubicin,^35^ higher MRD levels after induction,^37^ and a higher risk of relapse in the AYA population.^36^

Changes in drug resistance may also occur with age, resulting in reduced benefits from chemotherapy and poor outcomes in AYA patients. Leukemic cells from patients aged >10 years were significantly more resistant to prednisolone, dexamethasone, asparaginase, idarubicin, and 6-mercaptopurine compared with leukemic cells from children aged 1.5–10 years.^38^ Additionally, genetic factors specific to genes regulating the immune system, found in both pediatric and adult patients, have recently been shown to increase a patient's risk for developing an allergic reaction to asparaginase.^39^ Metabolism of certain chemotherapeutic drugs may also differ in adult patients, possibly increasing the incidence of drug-related toxicities or decreasing the effectiveness of critical chemotherapeutic agents, such as asparaginase or dexamethasone.^40^

## Treatment of AYA Patients with ALL

There are important differences in how ALL is treated in pediatric and AYA patients that may contribute to the poor outcomes observed in these patients. AYA patients are less likely to be enrolled in clinical trials. Clinical trials help to maintain standardization of care and may be an important factor toward ensuring the effectiveness of treatment.^10,41–43^ One American study found that <5% of AYA cancer patients were enrolled in clinical trials compared with 60% of pediatric patients.^44^ Additionally, children with ALL are generally cared for by a parent or legal guardian who may help maintain compliance throughout the prolonged treatment schedules. Treatment compliance is often problematic in AYA patients who may require substantial support to cope with treatment-related toxicities.^45–47^ Bhatia et al. evaluated adherence to mercaptopurine treatment schedules in 327 patients diagnosed with ALL.^43^ The results showed significantly lower adherence over a 6-month period in patients aged ≥12 years compared with younger patients (85.8% vs. 93.1%, *p* < 0.001). Investigators also found a higher risk of relapse in patients with lower adherence rates, highlighting the importance of high treatment adherence.^47^

While socioeconomic factors may contribute to the poor outcomes seen in AYA patients, likely the most critical—and most readily addressable—difference between pediatric and AYA treatment of ALL is the type of protocol used to treat each patient population. Pediatric protocols typically contain high doses of vincristine, corticosteroids, and asparaginase. In pediatric trials, adolescents are often treated as high-risk patients and are given a more intensified chemotherapy schedule. The treatment of AYA patients typically includes the use of allogeneic stem cell transplantation (SCT), a procedure associated with significant adverse events (AEs) and with a transplant-related mortality rate up to 20%. Pediatric protocols restrict SCT to a minority of patients with specific high-risk features.^48,49^

**FIG. 1.**
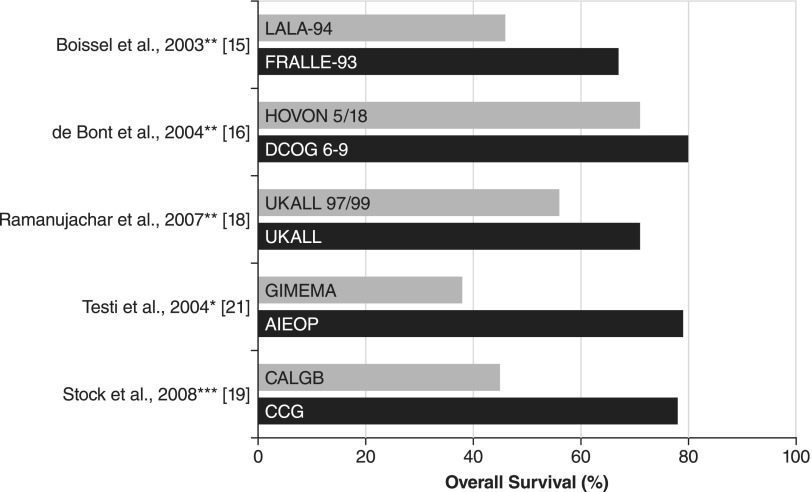
Retrospective comparison of outcomes in adolescent and young adult (AYA) patients treated on pediatric and adult protocols. *2-year OS; **5-year OS; ***7-year OS. AIEOP, Italian Association of Pediatric Hematology and Oncology; CALGB, Cancer and Leukemia Group B; CCG, Children's Cancer Group; DCOG, Dutch Childhood Oncology Group; FRALLE-93, French ALL Cooperative Group; GIMEMA, Italian Group for Adult Hematologic Diseases; HOVON, Hemato-Oncology Cooperative Study Group; LALA-94, Leucemie Aiguë Lymphoblastique de l'Adulte; OS, overall survival.

During the past decade, a number of retrospective trials have evaluated outcomes in AYA patients treated on either adult or pediatric protocols.^15–21^ The results of these studies show a consistent trend toward improved outcomes for AYA patients treated on pediatric ALL protocols compared with similar patients treated on adult ALL protocols (Fig. 1).^15,16,18,19,21^ Boissel et al. compared outcomes in patients aged 15–20 years treated according to the French ALL Cooperative Group (FRALLE)-93 and Leucemie Aiguë Lymphoblastique de l'Adulte (LALA)-94 protocols.^15^ The pediatric FRALLE-93 protocol included significantly more vincristine, steroids, and asparaginase compared with the adult LALA-94 protocol. In FRALLE-93, patients were exposed to up to 20 times more cumulative amounts of asparaginase compared with patients in LALA-94. Asparaginase was incorporated into induction and delayed intensification in FRALLE-93 compared with a 2-day exposure in LALA-94. Investigators found that the patients treated in the pediatric FRALLE-93 trial showed significantly higher complete response (CR, 94% vs. 83%), 5-year event-free survival (EFS; 67% vs. 41%), and OS (78% vs. 45%) compared with patients treated in the adult LALA-94 trial.^15^

The North American Cancer and Acute Leukemia Group B (CALGB) and the Children's Cancer Group (CCG) also retrospectively evaluated patients treated according to adult- or pediatric-based protocols.^19^ Overall, 321 AYA patients, aged 16–20 years, were treated on either adult CALGB or pediatric CCG treatment regimens. CALGB trials included a five-drug remission induction regimen (cyclophosphamide, daunorubicin, vincristine, prednisone, and asparaginase). Patients in the CCG trials were treated with either a standard CCG protocol or an augmented regimen that included greater exposure to asparaginase during consolidation, interim maintenance, and delayed intensification. At a 7-year follow-up, AYA patients in CCG showed EFS of 63% and OS of 67% compared with AYA patients in CALGB who showed EFS of 34% and OS of 46%. Subsequent retrospective reports revealed similar benefits when evaluating adolescent patients treated with pediatric protocols.^16–18^ Taken together, these results show a consistent trend toward better outcomes for young adolescent patients (aged 15–19 years) treated on pediatric regimens with greater cumulative doses of vincristine, steroid, and asparaginase.

Although these results highlight substantial improvements for adolescents treated on pediatric regimens, the patient populations in these retrospective studies were restricted to individuals aged 20 years and younger. Treatment-related AEs associated with asparaginase-containing regimens are generally believed to increase with age. Patients aged 46–60 years showed poor tolerance of asparaginase when compared with patients aged 14–45 years.^50^ Establishing the feasibility of asparaginase use in the older adult population is currently a critical area of research.

Results of several recent clinical trials suggest that the improved outcomes with pediatric-like treatment protocols can be generalized to the broader AYA population.^50–53^ Storring et al. evaluated 85 BCR-ABL–negative newly diagnosed ALL patients (aged 18–60 years) treated on a modified Dana–Farber Cancer Institute (DFCI) 91-01 Consortium Protocol, which included high-dose weekly asparaginase for 30 weeks during induction.^51^ The CR rate in these adult patients was 89%, and the 5-year OS was 63%. Age was a prognostic factor, with younger adult patients, aged 35 years or younger, showing greater 3-year OS compared with patients older than 35 years of age (83% and 52%, respectively). Prolonged asparaginase treatment was generally well tolerated, with only 12 patients failing to receive the complete 30 weeks of scheduled asparaginase therapy during intensification. Interestingly, patients who received at least 80% of their planned asparaginase therapy showed significantly greater 3-year OS and a lower cumulative risk of relapse compared with patients who were unable to receive >80% of their scheduled treatment. Fathi et al.^54^ examined the efficacy in an older population of a protocol derived from a completed DFCI consortium regimen used in younger adults. Pegylated (PEG)-asparaginase was given to 30 patients aged 51–75 years during induction and Cycle 1 of consolidation phases of therapy. A total of 19 of 29 evaluable patients achieved CR. Of the 18 patients who achieved CR after induction therapy, disease-free survival (DFS) at 1 year was 77%; the 1-year OS of 61% for evaluable patients with at least 1 year of follow-up was significantly higher than the historical rate of 33%.

The inclusion of asparaginase is potentially a key factor in the achievement of improved long-term outcomes for AYA patients treated on pediatric protocols. Hyper-CVAD is an asparaginase-free chemotherapeutic regimen widely used to treat adults with ALL.^13,55–61^ This regimen consists of two alternating cycles: Cycle A, characterized by fractionated cyclophosphamide, vincristine, doxorubicin, and dexamethasone, and Cycle B, characterized by high-dose methotrexate and cytarabine.^13,59^ A number of recent studies have retrospectively compared patients treated on pediatric-inspired and hyper-CVAD.^61–63^ In one study, patients treated on either the CALGB-8811 or hyper-CVAD treatment regimen were retrospectively compared.^61^ As reviewed above, CALGB-8811 included the use of asparaginase, although at a lower cumulative dose than in pediatric-based protocols. The investigators found that the majority of patients on both protocols achieved first remission, with CR rates of 84% for hyper-CVAD and 74% for CALGB-8811. However, a lower 5-year OS rate was seen in patients treated on hyper-CVAD compared with CALGB-8811 (26% vs. 44%, respectively). Similarly, a recent report from Alacacioglu et al.^62^ found similar CR rates after induction therapy for patients treated with BFM or hyper-CVAD protocols (95% and 96%, respectively). However, patients in the BFM group showed higher 5-year survival rates compared with the hyper-CVAD group (59% vs. 34%, respectively). In contrast with these reports, Rytting et al.^63^ found no significant difference in 3-year OS in a retrospective study of patients treated on an augmented-BFM protocol when compared with historically matched patients treated with hyper-CVAD (74% and 71%, respectively).^63^ Outcomes for clinical trials of AYA patients with ALL treated on pediatric-like and hyper-CVAD regimens are summarized in Table 1.^50,52,53,55,58–61,63–66^

**Table 1.** Characteristics and Outcomes in Older Patients Treated on Pediatric-Like and Hyper-CVAD Protocols for ALL

*Reference*	*Patients (*n*)*	*Age, years median (range)*	*CR*	*OS, years*	*OS*
*Pediatric-like*					
Ribera et al., 2008^53^	81	20.0 (15–30)	98%	6	69%
Huguet et al., 2009^50^	225	31.0 (15–60)	93.5%	3.5	60%
DeAngelo et al., 2007^64^	75	28 (18–50)	84%	2	77%
Rijneveld et al., 2011^65^	54	26 (17–40)	91%	2	72%
Haiat et al., 2011^52^	40	33 (18–55)	90%	3	75%
Cluzeau et al., 2012^66^	89	19 (15–29)	99%	5	66%
Rytting et al., 2014^63^	85	21 (13–39)	94%	3	74%
*Hyper-CVAD*					
Garcia-Manero et al., 2000^59^	204	39.5 (16–79)	91%	5	<30 years = 54%; 30–49 years = 42%
Kantarjian et al., 2004^58^	288	40 (15–92)	92%	5	38%
Kozlowski et al., 2014^60^	24	32 (18–72)	89%	5	47%
Morris et al., 2011^55^	63	29 (14–76)	86%	5	48%
Buyukasik et al., 2013^61^	166	29 (16–63)	84.2%	5	26.3%

ALL, acute lymphoblastic leukemia; CR, complete response; OS, overall survival.

Similar long-term results were observed in a modeling study of AYA patients treated on hyper-CVAD or a pediatric-inspired protocol.^67^ Investigators used an exploratory decision-analytic approach to model the survival and quality-adjusted survival of AYA patients treated according to these different protocols. Model results at 1 year showed only minor differences between protocols. However, survival and quality-adjusted survival were both noticeably greater in the pediatric-inspired group at 5 and 10 years compared with hyper-CVAD.

## Asparaginase

The use of asparaginase as a chemotherapeutic agent dates back to 1953 when Kidd et al. reported that guinea pig serum induced leukemic cell death when injected into diseased mice.^68^ In 1961, asparaginase was identified as the agent responsible for this antileukemic effect and was subsequently investigated for use in human cancer patients.^69^ Unlike healthy cells, leukemic blasts do not produce asparagine and rely exclusively on extracellular sources of the amino acid.^70^ Asparaginase takes advantage of this metabolic difference by catalyzing the breakdown of asparagine to aspartic acid and ammonia, depriving leukemic cells of the amino acid.^71^ Healthy cells continue to produce asparagine de novo, through the enzymatic action of asparagine synthetase, and are largely spared. However, prolonged asparagine deprivation in leukemic cells results in reduced DNA, RNA, and protein synthesis and eventually leads to the activation of programmed cell death mechanisms.^71^

Asparaginase activity levels show an inverse relationship with circulating asparagine concentrations and are often used to determine a patient's asparagine depletion status.^72–75^ Human and animal studies have shown that serum asparaginase activity levels ≥0.1 IU/mL are adequate for asparagine depletion, and this criterion has become accepted as the minimal therapeutic level in practice.^74,76^ The regular measurement of asparaginase activity was used in several pediatric trials to ensure that the patients maintain asparagine depletion throughout treatment.^77,78^

As a foreign protein, asparaginase has the potential to elicit an immune response when administered to patients. Immune reactions to asparaginase are broadly classified as clinical hypersensitivity or subclinical hypersensitivity (also referred to as “silent inactivation”). Both clinical and subclinical hypersensitivity are associated with reduced asparaginase activity levels in patients and can lead to poor outcomes if not properly addressed.^78–81^

There are three types of asparaginase approved for the treatment of ALL. Native *Escherichia coli* asparaginase and PEG-asparaginase are derived from the bacterium *E. coli*.^82^ Owing to their common origins, these asparaginases display a significant amount of cross-reactivity with respect to their ability to elicit an immune response in patients.^83^ The third asparaginase, asparaginase *Erwinia chrysanthemi*, is derived from the bacterium *Erwinia chrysanthemi* (revised taxonomy: *Dickeya dadantii*) and shows limited cross-reactivity with *E. coli–*derived asparaginases.^83,84^ Asparaginase *Erwinia chrysanthemi* is indicated as a component of a multiagent chemotherapeutic regimen for the treatment of patients with ALL who have developed hypersensitivity to *E. coli*–derived asparaginase.^85^ Recently, the supply of native *E. coli* asparaginase has been discontinued in the United States, and has largely been replaced by PEG-asparaginase as first-line treatment for patients with ALL.^86^

All three asparaginases share the same mechanism of action, the deamination of asparagine. However, each asparaginase has markedly different pharmacokinetics, which must be accounted for when establishing dose and treatment schedules (Table 2).^87^ Due to the addition of polyethylene glycol, PEG-asparaginase shows a longer half-life compared with the other two asparaginase preparations and is therefore able to provide a longer period of asparagine depletion from a single dose.^79,88^ Asparaginase *Erwinia chrysanthemi* shows a more rapid half-life and should be administered more frequently than PEG-asparaginase to maintain asparagine depletion.^87,89^ The recommended substitution dose of asparaginase *Erwinia chrysanthemi* in patients who experience hypersensitivity to PEG-asparaginase is 25,000 IU/m^2^ administered intravenously (i.v.) or intramuscularly (i.m.) six times on a Monday, Wednesday, Friday schedule (2 weeks) to replace each remaining dose of PEG-asparaginase in the patients' scheduled treatment.^85,89^ In 2014, the U.S. Food and Drug Administration approved the i.v. administration of asparaginase *Erwinia chrysanthemi*.^85^ Based on population data, the mean half-life of asparaginase *Erwinia chrysanthemi* following i.v. infusion was 7.51 hours, compared with 15.6 hours following i.m. injection.^85^ Due to these differences in pharmacokinetics, it is suggested that nadir serum asparaginase activity (NSAA) levels be monitored in patients receiving i.v. asparaginase. Patients may be switched to i.m. administration if desired NSAA levels are not achieved.^85^

**Table 2.** Half-Life of the Three Asparaginase Preparations

	*Asparaginase* Erwinia chrysanthemi*^79^ 1 × i.m. dose of 25,000 IU/m^2^ (*n* = 10)*	*Asparaginase* Erwinia chrysanthemi*^85^ 1 × i.v. dose of 25,000 IU/m^2^ (*n* = 24)*	*Native* Escherichia coli *asparaginase^a,79^ 1 × i.m. dose of 25,000 IU/m^2^ (*n* = 17)*	*PEG-asparaginase^79^ 1 × i.m. dose of 2500 IU/m^2^ (*n* = 10)*
Half-life, days (mean ± SD)	0.65 ± 0.13	0.31 ± 1.79	1.28 ± 0.35	5.73 ± 3.24

^a^ Native *E. coli* asparaginase is no longer available in the United States.

i.m., intramuscular; i.v., intravenous; PEG, polyethylene glycol (pegylated); SD, standard deviation.

## Asparaginase Use in AYA Patients

The pharmacokinetics of asparaginase has been extensively studied in pediatric patients. However, few investigations have focused on asparaginase use in AYA patients.^90–92^ Douer et al. evaluated asparaginase pharmacokinetics in adult patients (aged 17–55 years) with ALL.^90^ Twenty-five newly diagnosed patients were administered a single dose of PEG-asparaginase 2000 IU/m^2^ during induction. In these adult patients, the population average asparaginase activity correlated well with serum asparagine deamination, and the half-life of i.v. administered PEG-asparaginase was reported as 7 days, similar to what has been shown in pediatric patients.^87,92^ Furthermore, asparaginase activity following PEG-asparaginase administration was long-lasting, with 81% of patients showing complete deamination of asparagine at 21 days post-infusion.

Although it is not known how long circulating asparagine levels must be depleted for programmed cell death mechanisms to be activated in leukemic blasts, there is strong clinical evidence that prolonged exposure to asparaginase is associated with superior outcomes.^93,94^ The DFCI ALL Consortium Protocol 91-01 evaluated outcomes in 352 pediatric patients (aged 0–18 years) treated on an intensive 30 weeks of high-dose asparaginase during intensification.^93^ The investigators found an increased 5-year EFS in patients who were able to receive >25 weeks of asparaginase therapy. EFS was 90% for patients who received >25 weeks of asparaginase compared with 73% in patients who tolerated ≤25 weeks (*p* < 0.01). Common reasons for discontinuing asparaginase treatment prior to the 25-week mark in this study included pancreatitis (39%), clinical allergy (19%), and central nervous system thrombosis (12%).

The CALGB-9511 study measured asparaginase activity in 85 adult patients (aged 17–71 years) receiving PEG-asparaginase 2000 IU/m^2^ during induction and first intensification.^91^ Depletion was defined as trough serum asparaginase activity >0.03 IU/mL in this study. The majority of adult patients maintained asparagine depletion (*n* = 63), with 22 patients failing to achieve asparaginase activity >0.03 IU/mL in at least one measurement. Adult patients who failed to achieve depletion showed significantly inferior OS and DFS compared with patients with depletion. The reported hazard ratio for OS and DFS between these two groups of patients was 2.37 (*p* = 0.002) and 2.21 (*p* = 0.012), respectively.

## Safety and Toxicity of Asparaginase in AYA Patients

Asparaginase use in AYA patients has historically been limited because of the perception of an increased risk of toxicity with age. Hepatotoxicity—grade 3–4 elevations in aspartate aminotransferase and alanine aminotransferase—was the most common toxicity in a study with 152 patients aged 18–60 years given PEG-asparaginase according to a regimen adapted from the augmented arm of the CCG-1882 protocol.^95^ In an evaluation of 76 newly diagnosed adolescent and adult patients (aged 14–68 years) treated with PEG-asparaginase, investigators reported a greater frequency of grade 3–4 hepatic and pancreatic AEs compared with 1274 pediatric patients.^96^ The authors suggest that coexisting morbidities, possibly due to increased use of hepatotoxic and pancreatotoxic drugs, may contribute to the increased toxicities seen in AYA patients, as healthy adults were able to tolerate PEG-asparaginase to an extent comparable with younger patients in the study.^96^ Although increased pancreatic toxicity with asparaginase is often associated with AYA patients, it should be noted that several studies reported a relatively low rate of pancreatitis in AYA patients undergoing asparaginase treatment.^50,53,65,95,97^ Detailed recommendations regarding the management of pancreatitis and other asparaginase-associated toxicities are beyond the scope of this review and have been summarized elsewhere.^96^ The continued use of asparaginase is not recommended in the event of clinically confirmed pancreatitis.^96^

Asparaginase use has also been associated with venous thromboembolic events (VTE) in both pediatric and adult patients. Decreased synthesis of antithrombin and fibrinogen, leading to increased thrombin generation, is commonly associated with asparaginase use.^98–100^ Asparaginase-related VTE has been reported in 3–5% of pediatric patients undergoing treatment for ALL.^99,101–104^ Increased age has been shown to be a significant predictor of VTEs, with thrombotic complications occurring in up to 34% of adult patients in one study.^105^ Deep venous thrombosis should be managed with anticoagulation therapy, and it is recommended that asparaginase be temporarily discontinued in the case of clinically significant bleeding or thrombotic events.^96^ Many antithrombotic prophylactic approaches have been adopted, including heparin or antithrombin substitution, with varied clinical evidence.^102,105,106^ Asparaginase may be reintroduced once acute toxicity and clinical symptoms have resolved.^96^

Douer et al.^107^ evaluated the pharmacokinetics and toxicity of PEG-asparaginase in 51 adult patients (aged 18–57 years) during induction therapy. In this trial, PEG-asparaginase dosing was structured to avoid overlapping toxicity with other chemotherapy drugs and to improve overall treatment tolerance. Additionally, PEG-asparaginase intervals were lengthened to ≥4 weeks. The investigators reported that the most common asparaginase-associated toxicities (grade 3–4) were hyperbilirubinemia, transaminitis, hyperglycemia, and hypertriglyceridemia (Table 3). No deaths were reported as a result of asparaginase-related toxicity. Overall, PEG-asparaginase was discontinued owing to prohibitive toxicity in 20% of patients (*n* = 10). The most common reasons for discontinuation in these patients were pancreatitis (*n* = 6) and severe clinical hypersensitivity (*n* = 3).

**Table 3.** PEG-Asparaginase Toxicity in Patients Aged 18–57 Years (*N* = 51)^107^

	*Grade 1 or 2*	*Grade 3 or 4*	
*Toxicity*	n	n	*%*
Pancreatitis	0	7	13.7
Allergy	2	3	5.9
Deep venous thrombosis	0	8	15.7
Bleeding	5	1	2
Transaminitis	19	32	62.7
Hyperbilirubinemia	30	16	31.3
Hyperglycemia	29	17	33.3
Hypertriglyceridemia	6	9	17.6
Fatigue	34	4	7.8
Neuropathy	10	1	2
Vomiting	19	2	3.9
Nausea	34	3	5.9
Diarrhea	16	0	0
Constipation	18	0	0
Headache	27	1	2
Edema	6	0	0

Reprinted and adapted with permission from Douer et al. 2014.^107^ © 2014 American Society of Clinical Oncology. All rights reserved.

Clinical hypersensitivity reactions range in severity, from a localized rash or pain around the injection site to a systemic immune reaction, possibly resulting in anaphylaxis.^108^ The prevalence of asparaginase hypersensitivity in pediatric patients with ALL ranges from 3–45%.^75,78,81,97,109,110^ Although less clinical data exist in adult patients, hypersensitivity rates do not appear to differ with age. In CALGB-8811, the incidence of severe hypersensitivity reactions was reported as 11% for adults treated with native *E. coli* asparaginase.^111^ For newly diagnosed adult patients (aged 15–39 years) enrolled in the U.S. Intergroup Trial C10403, grade 3–4 hypersensitivity reactions were found to occur in 8–13% of patients treated with PEG-asparaginase.^112,113^ In the compassionate-use trial for asparaginase *Erwinia chrysanthemi*, hypersensitivity reactions were reported in 11% of AYA patients compared with 15% of patients aged 10 years and younger.^97^ Patients with hypersensitivity to *E. coli*–derived asparaginase should immediately discontinue therapy and be switched to treatment with asparaginase *Erwinia chrysanthemi*. Asparaginase *Erwinia chrysanthemi* is indicated for use as a component of a multiagent chemotherapeutic regimen for the treatment of patients with ALL who have developed hypersensitivity to *E. coli*–derived asparaginase.^80,82^

The development of antiasparaginase antibodies can occur in patients without any overt signs of an immune response, a condition referred to as subclinical hypersensitivity or “silent inactivation.”^78,81,114^ Subclinical hypersensitivity in pediatric patients is characterized by decreased asparaginase activity levels and can be associated with poor outcomes in those who have not switched to an alternative asparagine preparation once subclinical hypersensitivity is established.^77,78,81^ Asparaginase *Erwinia chrysanthemi* shows no cross-reactivity with *E. coli*–derived asparaginases and is the preferred alternative in patients who develop allergy to native *E. coli* or PEG-asparaginase.^76,83,96^ The regular monitoring of asparaginase activity levels throughout the course of therapy can help identifiy patients who develop subclinical hypersensitivity and allows clinicians to adjust treatment actively to the individual patient.^78^ In a study by Burke et al., patients aged 18–57 years were treated with PEG-asparaginase according to a regimen adapted from the pediatric protocol CCG-1882.^115^ A total of 34 of 61 PEG-asparaginase doses were associated with antiasparagainse antibody production, 27 of which occurred within the first 7 days post-dose, and only two of which were associated with an overt hypersensitivity response. However, serum asparaginase activity <0.2 IU/mL at 14 days post-dose was found in only 2 of 26 antibody-positive patients, suggesting that antibody production is not associated with increased drug clearance in these patients.

**FIG. 2.**
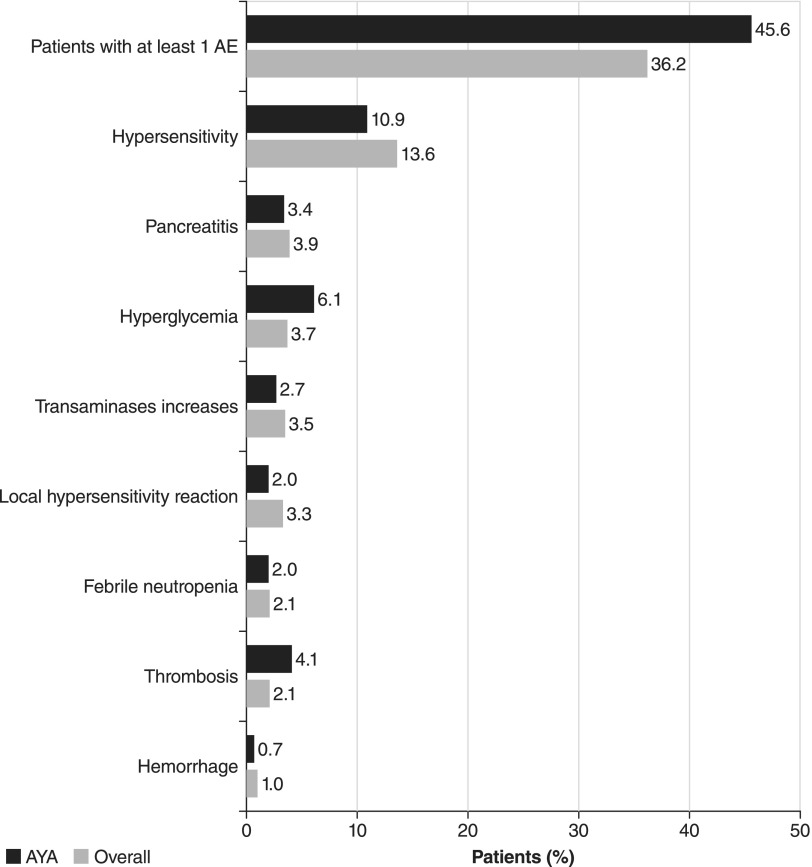
Comparison of adverse events (AEs) in AYAs (*n* = 147) with the overall patient population (*n* = 940) in a compassionate-use trial of asparaginase *Erwinia chrysanthemi*.^97^

A large (*n* = 1368) compassionate-use trial evaluated the safety and toxicity of asparaginase *Erwinia chrysanthemi* in patients of various ages.^97^ The study included 147 AYA patients (aged 16–39 years) and reported that the safety profile of asparaginase *Erwinia chrysanthemi* in these patients was consistent with that found in the total population (Fig. 2). The reported incidence of pancreatitis was similar for patients aged 16–40 years and younger than 10 years (3.4% and 3.0%, respectively). Rates of thrombosis were slightly higher in AYA patients, with thrombosis reported in 4.1% of AYA patients compared with 1.3% of children younger than 10 years of age. All patients included in the study had a previous grade ≥2 hypersensitivity reaction to *E. coli*–derived asparaginase and were switched to asparaginase *Erwinia chrysanthemi* 25,000 IU/m^2^ administered on a Monday, Wednesday, Friday regimen. Overall, asparaginase *Erwinia chrysanthemi* was well-tolerated in AYA patients and allowed the majority (72.8%) to complete their planned course of asparaginase treatment. Although outcomes were not reported in this study, the therapeutic benefits of receiving the full course of asparaginase treatment are well-established.^97^

Safety and toxicity data from a large prospective trial evaluating AYA patients treated on a pediatric regimen were recently presented in abstract form.^112,113^ Between November 2007 and August 2012, 318 patients (aged 16–39 years) were enrolled in the U.S. Intergroup Trial C10403 and treated according to the Children's Oncology Group (COG AALL0232) protocol by adult oncologists. Safety and toxicity results were compared with 159 patients from COG AALL0232. The incidence of hepatic toxicity, pancreatitis, and osteonecrosis were found to be similar between AYA patients in both groups. Grades 3–4 hepatic and pancreatic dysfunction were slightly greater in C10403 compared with pediatric patients, likely due to an increased use of alcohol or other hepatotoxic and pancreatotoxic drugs. Overall, healthy adults were able to tolerate asparaginase therapy comparable with younger patients. Treatment-related mortality was low (2%) in C10403, and the investigators concluded that treatment with a pediatric regimen was feasible in AYA patients up to 40 years of age.

## Conclusions and Future Directions

The treatment of AYA patients diagnosed with ALL represents a unique challenge to the clinical oncologist. Substantial evidence indicates that AYA patients treated on pediatric protocols show improved survival compared with patients treated on traditional adult regimens. Although certain treatment-related toxicities are more prevalent in AYA patients, emerging clinical evidence suggests that high-intensity pediatric regimens are feasible in the AYA population. As is the case in pediatrics, the effective management of treatment-related toxicities is critical to ensure that AYA patients receive the full benefit of ALL therapy. The ongoing development of novel asparaginase preparations, such as pegylated recombinant *Erwinia*-derived asparaginase (pegcrisantaspase) and red blood-cell encapsulated asparaginase, also promises to reduce immunogenicity and increase the overall length of asparagine depletion.
